# Identification of Metal Contamination Sources and Evaluation of the Anthropogenic Effects in Soils near Traffic-Related Facilities

**DOI:** 10.3390/toxics9110278

**Published:** 2021-10-21

**Authors:** Hong-gil Lee, Yoon Joo Byun, Young-Woo Chun, Hoe-Jung Noh, Dong-Jin Kim, Hyun-Koo Kim, Ji-In Kim

**Affiliations:** Soil and Groundwater Research Division, National Institute of Environmental Research, 42 Hwangyoung-ro, Incheon 22689, Korea; lhg7090@korea.kr (H.-g.L.); yju0902@korea.kr (Y.J.B.); marvelwoo@korea.kr (Y.-W.C.); hjnoh99@korea.kr (H.-J.N.); gimdj83@korea.kr (D.-J.K.); khk228@korea.kr (H.-K.K.)

**Keywords:** metal contamination, anthropogenic contamination, urban traffic, heavy metal

## Abstract

Traffic-related facilities typically have much lower metal emissions than other sources; however, they can be numerous and widespread as well. Subdividing pollution sources is necessary to assess soil contamination characteristics and identify sources according to the contamination cause. Anthropogenic contamination by metals was quantitatively determined using contamination factor (Cf) and evaluated using multivariate analysis. More than half of the concentrations for Zn, Pb, and Cu in soils were higher than that in the natural background (NB). Cf of metals was, in decreasing order, Zn > Pb = Cu > Ni = As. Zn, Pb, and Cu were identified as anthropogenic contaminants in correlation analysis. Principal component analysis showed that the two main contamination causes were coarse particles from the maintenance or crushing activities of vehicles and nonexhaust/exhaust emissions. Clusters were classified according to those two anthropogenic and lithogenic causes and included Group I (Zn, Pb, and Cu in garages, auto repair shops, and auto salvage yards), Group II (Zn, Pb, and Cu in parking lots, driving schools, and roadsides), and Group III (As and Ni with high lithogenic properties). Anthropogenic input and sources of soil contamination by metals in traffic-related facilities were appropriately estimated through the combination of Cf and multivariate analysis.

## 1. Introduction

Traffic-related facilities refer to places for vehicle movement and maintenance, such as roads, repair shops, and garages, where contamination may occur locally or in a wide area by many contaminants. These facilities typically have much lower emissions but can be numerous and widespread as well [1,2]. Vehicular traffic and, by implication, road surfaces are among the most important sources of pollutants to the environment [3]. There have been various studies on the pollutants that may be generated in traffic-related facilities [4,5,6,7,8,9,10]. Various metals are discharged from automobile emissions, such as vehicles’ used oils, worn parts, used batteries, and fuel additives including trace amount of organic and inorganic chemicals, thereby facilitating soil contamination by metals [11,12]. These metals enter human bodies and other ecosystems, such as groundwater, rivers, the atmosphere, and crops, through bioaccumulation in food chains [13,14,15]. Some metals are essential sources of nutrients for living beings; however, they are generally considered toxic to humans and animals, even at low concentrations, because of their carcinogenic effects [16,17,18,19,20].

Pollution indices (PIs) are techniques to quantify the contamination level of sub-stances of interest in soils by calculating the ratio of the concentration discovered in a site suspected of pollution to the reference concentration (e.g., natural background (NB) or the concentration of a specific element on earth’s crust) [21]. They are used to evaluate the pollution level of various environmental media, such as soil, water, and sediments [22,23,24,25]. Because the contamination factor (Cf) was based on the standardization of a tested element against a reference one, the accuracy of multivariate analysis on soil contamination can be improved. Principal component analysis (PCA) and cluster analysis (CA) are used in the evaluation of anthropogenic contamination factor (Cf) of metals in soils and in the identification of contamination sources [26,27,28].

Although some studies have been conducted on the soil contamination impact of a traffic-related facility limited to a specific city or road in Korea, few studies have been conducted on various traffic-related facilities nationwide [29,30,31,32,33,34]. The Surveys of the Actual State of Soil Contamination (SASSC) are the national survey projects that examine regions with a high possibility of soil contamination considering soil contamination sources; the goal of these projects is to prevent hazards on citizens’ health and the environment by soil contamination. Traffic-related facilities are considered to be a source of soil contamination in SASSC and are analyzed every year by local governments, who check whether their contamination exceeds the standard limit. Thus, it is necessary to investigate the contamination characteristics, contamination causes, and a correlation between contamination sources and contaminants to provide a measure to control contamination sources. In this study, we (i) distributed the concentrations of metals associated with anthropogenic sources, (ii) evaluated the level of the anthropogenic contamination effect of metals, (iii) evaluated a correlation between metals and each contamination source, and (iv) categorized the contamination sources according to the contamination causes through PCA and CA.

## 2. Materials and Methods

### 2.1. Study Area, Sample Collection, and Analysis

From 2015 to 2019, 2330 soil samples were collected from car dismantling workshops (for recycling), repair shops, parking lots, car schools, garages and their adjacent roadsides throughout the nation. The contamination sources were classified into auto repair shops (ARS), auto salvage yards (ASY), driving schools (DSC), garages (GAR), parking lots (PAL), and roadside (RDS) according to the use of the facility. Port-related facilities and aviation-related facilities were excluded from the evaluation because they exhibited a regional distribution. A summary of the study area is described in Table S1 and Figure S1. Topsoil (0–15 cm) samples were used for consistent analysis of the contamination phenomenon. It has been known that urban surface soils, including those in the roadside and transportation industries, are indicators of heavy metal contamination from atmospheric deposition [35,36,37]. Five to ten soil samples in a region were mixed together to prepare a composite soil sample. All samples were decomposed using aqua regia after air drying and sieving (10–100 mesh). The decomposed solution was analyzed with AAS or ICP-AES considering the concentration level of the contaminant to be analyzed and available instruments at each local government. The method detection limit and detection limit (DL) were derived using reference materials before sample analysis begins and were satisfied with the goals of quality control.

In environmental data, data below the DL can be statistically substituted in a variety of ways [38,39,40]. In this study, it was substituted to DL/2 of each metal to avoid the underestimation of the Cf. The DL for each item is as follows: zinc (Zn), 1.0 mg kg^−1^; lead (Pb), 1.5 mg kg^−1^; copper (Cu), 1.0 mg kg^−^^1^; arsenic (As), 0.10 mg kg^−1^; and nickel (Ni), 0.4 mg kg^−1^.

### 2.2. NB Concentrations and Data Selection

The NB concentration data of metals in the National Institute of Environmental Research were used [41,42]. The NB areas are unpolluted natural/undisturbed sites which can represent the geological units in Korea (Table S2). The NB levels were as follows: zinc (Zn), 54.3 mg kg^−1^; lead (Pb), 18.4 mg kg^−1^; copper (Cu), 15.3 mg kg^−^^1^; arsenic (As), 6.83 mg kg^−1^; and nickel (Ni), 17.7 mg kg^−1^.

801 samples were selected as contaminated samples that have the upper confidence limit of 95 percentile of the NB as per the UK and Netherlands methods. Although these methods are somewhat arbitrary, it can guarantee the variability of most NB concentrations, except for extreme values [43,44,45].

### 2.3. Contamination Factor

Cf allows the assessment of soil contamination based on the concentrations of contaminants referenced to background levels of elements, as presented in Equation (1). The NB concentrations described in Section 2.2 were applied as background levels instead of the pre-industrial levels of metals in sediments suggested by Hakanson (1980) [46].

(1) $$\mathrm{Cf}=\frac{\mathrm{Measuredconc}.\;\mathrm{ofelement}}{\mathrm{Backgroundconc}.\;\mathrm{ofelement}}$$

Cf values (unitless) fall into the following four classes: no/low contamination (Cf < 1), moderate contamination (1 ≤ Cf < 3), considerable contamination (3 ≤ Cf < 6), and very high contamination (Cf ≥ 6).

### 2.4. Statistical and Multivariate Analysis

The data were statistically analyzed using the R package (version 4.0.2). Since the Shapiro–Wilk test results showed that all data had no normality, a non-parametric analysis significance test was conducted (Table S3). To test if there was a significant difference in variance among data, the Kruskal–Wallis method followed by the post-hoc Dunn test was used. Kendall’s correlation was used to analyze the correlation between metal concentrations in each contamination source. The analysis was conducted such that if τ (tau) was larger than 0.3, there was a positive correlation, and if the significant level satisfied the 95% significance level (*p* < 0.05), the correlation was significant. For the PCA among multivariate analyses, the “prcomp” function was used. The Kaiser–Meyer–Olkin (KMO) test was conducted to evaluate the usefulness of the dataset for PCA, and the criterion of the usefulness was set to >0.5. For the test of variance homogeneity for non-normal distributions, the Fligner–Killeen test was employed. The absolute loading of each component from the extracted PC was expressed to strong positive loading when its value was more than 0.4. For the hierarchical CA, the Ward algorithmic method and squared Euclidean distance were applied. The data and NB data for each contamination source used in the CA were re-adjusted to 0–100 percentiles for each contamination source to overcome the data size difference.

## 3. Results and Discussion

### 3.1. Distribution of Metals in Soils Nearby Contamination Sources

The concentration distributions of Zn, Pb, and Cu in the soils showed a significant difference according to the contamination source, whereas the concentration distributions of As and Ni had no significant difference in all contamination sources (Tables S4 and S5). The median values (mg kg^−1^) of the contaminant showed various distributions according to the contamination source: Zn 89.4 (RDS)–128.1 (DSC); Pb 19.3 (PAR)–31.4 (DSC); Cu 21.3 (PAR)–29.1 (ASY); As 2.6 (DSC)–5.2 (ASY); Ni 8.7 (RDS)–16.6 (DSC) (Table 1). The median values of Zn, Pb, and Cu were 1.7–2.3 times, 1.2–2.0 times, and 1.6–2.2 times higher than the median values of the NB. In particular, the median value of Zn exhibited the highest contamination level, as it exceeded the maximum value of the NB except for the median values of PAL and RDS.

Exhaust emissions from the fuel combustion of vehicles and nonexhaust emissions such as tire wear, brake wear, and road abrasion may occur in all contamination sources [47,48]. In addition, a number of studies reported that the average concentrations of Zn, Pb, and Cu in soils near RDS, ARS, and GAR were higher than the average background and control concentrations [4,7,49,50,51,52,53]. Tens to hundreds ppb of Zn, Pb, Cu, Ni, and As may be contained in gasoline and diesel [47,54,55]. Zn was contained in a range of 670–1760 mg kg^−1^ in motor engine oils with an engine wear prevention additive, zinc-dialkyl-dithio-phosphate [56,57,58]. Tire wear, brake wear, and asphalt road abrasion were also among the contamination sources of metals (including Zn, Pb, and Cu) that may be generated during vehicle driving and braking [48,59,60,61,62,63], and this result indicated that the load of pollutants onto soils increased the concentration of metal contaminants in soil. Thus, the high concentrations of Zn, Pb, and Cu in the soils were found as the results of their accumulation on surface soils by automobile-related emissions.

The median values of As and Ni showed a range of 2.6 mg kg^−1^ (DSC)–5.2 mg kg^−1^ (ASY) and 8.7 mg kg^−1^ (RDS)–16.6 mg kg^−1^ (DSC), respectively, which were relatively lower than the median values of As and Ni of the NB (Table 1). A small amount of As has been used for Pb component enhancement in car batteries, and As has been known to be present in brake and fuel [64,65]. Ni could be emitted from tire wear and motor/engine oil spills [65,66,67,68]. However, Ni and As concentrations contained in the fuel, tires, and brakes were considerably lower than that of Zn, Pb, and Cu. Several studies on contamination sources in urban areas reported that As/Ni concentrations in soils was lower than that of other metals or the background level proposed in their studies, including the lithogenic properties [49,69,70,71]. The effect of As and Ni soil deposition in soil near the traffic-related facilities was considered lower than Zn, Pb, and Cu deposition.

### 3.2. Contamination Factor

**Figure 1:**
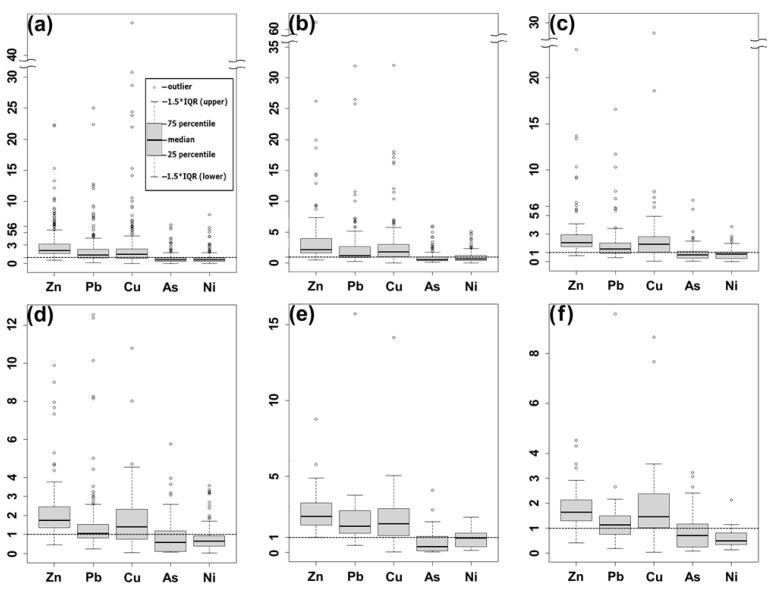
Contamination factors of metals in selected contamination sources; (**a**) garages (GAR), (**b**) auto repair shops (ARS), (**c**) auto salvage yards (ASY), (**d**) parking lots (PAL), (**e**) driving schools (DSC), (**f**) roadside (RDS).

CfZn, CfPb, and CfCu in soils near GAR, ARS, ASY, and DSC showed moderate to very high level of contamination in more than 2/3 of the total surveyed area for each contamination source, which verified the anthropogenic contaminations by Zn, Pb, and Cu (Figure 1a–c,e). CfAs and CfNi in the soils showed no or low levels of contamination in around up to 3/4 of the samples, which revealed lower anthropogenic effects than that of CfZn, CfPb, and CfCu. The contamination level of Zn was evaluated as the most severe in all contamination sources, and the Cf values were, in decreasing order, Zn > Pb ≒ Cu > As ≒ Ni. The highest Cf values in the contamination sources were as follows: Zn 62.1 in ARS; Cu 47.0 and 28.7, respectively, in GAR and ASY; Pb 15.7 in DSC.

Over half of the soil samples in PAL and RDS demonstrated moderate to very high levels of contamination for Zn, Cu, and Pb, while CfAs and CfNi showed no or low levels of contamination at more than 69% of soil samples from those sources (Figure 1d,f). The Cf values were, in decreasing order, Zn > Cu > Pb > As > Ni. The highest CfAs and CfNi were 5.8 and 3.6 at PAL (considerable to very high levels of contamination), and 3.2 and 2.1 at RDS (moderate to considerable levels of contamination). Their Cfs were lower than that of CfZn, CfPb, and CfCu (very high levels of contamination).

### 3.3. Correlation Analysis

Positive correlations among CfZn, CfPb, and CfCu were found in the GAR, ARS, and ASY soils (*p* < 0.005) (Table 2). The Zn, Pb, and Cu concentrations in soils of ASY were higher than those of the background site, in which auto-related parts were presumed as major contamination sources [8]. Zn, Pb, and Cu are the most common metals released from automobiles, accounting for at least 90% of the total metals in road runoff [60]. Thus, Zn, Pb, and Cu were anthropogenically accumulated in the soil via vehicle-related emissions during automobile repair or salvage processes.

CfNi in the soil among the three contamination sources had a positive correlation with CfCu and CfZn, CfCu and CfPb, and CfCu. Tires and brakes in vehicles contain not only Zn, Pb, and Cu but also Ni [72]. Therefore, the correlation of Ni with other metals was caused by the different loading of nonexhaust particles according to the vehicle types and conditions. This correlation can also be verified from several studies, in which the concentrations of Ni, Zn, Pb, and, Cu in soils near auto-mechanic shops, salvage yards, and electronic waste treatment plants showed significant decreases depending on the horizontal distance from the contamination sources [73,74,75,76]. CfAs in the soil among the three contamination sources showed a similar correlation as that of the metals, but its correlation coefficient was lower than that of other elements (τ < 0.3). This tendency seemed to be the result of arsenic usages in automobile-related parts, but its amount was considerably lower than that of other metals.

Correlations between CfZn-CfPb, CfZn-CfCu, and CfNi-CfCu in PAL were positive (*p* < 0.005). In addition, a positive correlation between CfCu-CfPb was found, but it was statistically weak (τ < 0.3) (Table 2). Not only was the average concentration of Zn, Pb, and Cu in the soils of the parking lot and trailer park in use higher than their average background concentration, but also, the coefficient of variance was more than around twice that of other elements, and the contamination in the parking lot soil was assumed to be caused by parts of different vehicles [7,10]. In addition, the metal amount in the road dust per unit area in the parking lot was as follows: Zn > Pb > Cu > Ni > As [51]. Therefore, the correlation between metal concentrations found in the parking lot soils might be determined by the vehicle type.

There was a positive correlation between CfZn-CfPb, CfPb-CfCu, CfAs-CfCu, and CfNi-CfCu in DSC (*p* < 0.01) (Table 2). Generally, a regular number of vehicles are continuously driven in DSC, and a land boundary is fenced by walls. Nonexhaust or exhaust emissions by vehicles are not easily diffused in the atmosphere and have more chances to deposit in the land. As a result, the correlation between metal concentrations was higher than that of PAL and RDS.

In RDS, there was a positive correlation between CfPb-CfCu and between CfPb-CfAs, but the correlation coefficient between CfPb and CfCu was lower than that in GAR, ARS, and ASY (Table 2). This was because RDS is mainly located near the expressways, and the distance between road and soil was sufficiently more than that of other contamination sources, which made it difficult for contaminants to reach the soil. Studies by Akbar et al. (2006) [9] and Nabulo et al. (2006) [77], also supported this hypothesis that as the distance between the road and contamination source increased, the metal concentration decreased, and the concentration of metals was observed at the level of NB at around 30 m distance from the road [9,77]. That is, the metal accumulation level in the soil around the road depends on the distance between the road and contamination source.

### 3.4. Multivariate Analysis

#### 3.4.1. Principal Component Analysis

PCA results for all pollution sources were considered as useful (KMO values 0.54 to 0.78), and the contamination level of each metal at specific sources had no equality of variances (*p* < 0.005) (Table S6). Two components (PC1 and PC2) whose standard deviation was the largest in all contamination sources were extracted (Table S7).

##### Garage, Auto Repair Shop and Auto Salvage Yard

**Figure 2:**
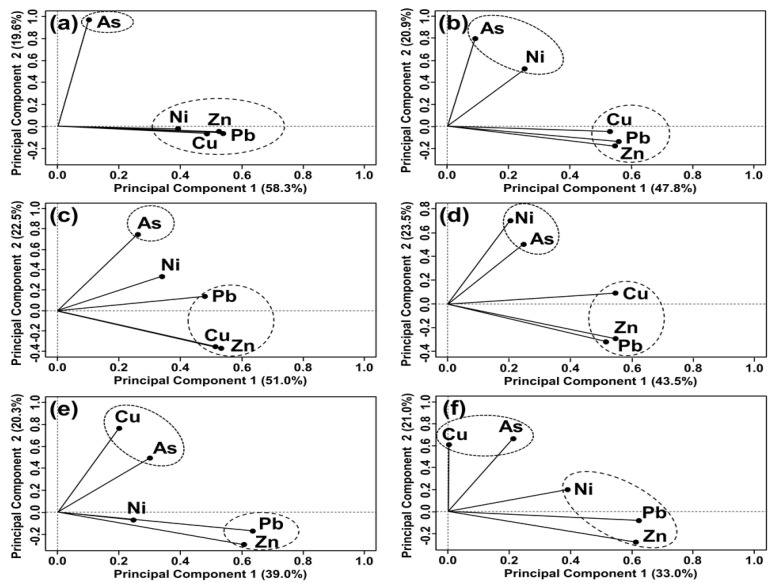
Extracted principal components (PCs) in selected contamination sources: (**a**) garage (GAR), (**b**) auto repair shop (ARS), (**c**) auto salvage yard (ASY), (**d**) parking lot (PAL), (**e**) driving school (DSC), and (**f**) roadside (RDS). The dotted lines represent the xy axis.

In GAR, ARS, and ASY, the first components (PC1) are responsible for 47.8–58.3% of the total variance and significant positive loadings are shown for Zn, Pb, and Cu (Figure 2a–c; Table S8). Because mainly vehicle maintenance or dismantling rather than vehicle driving occurred in these sources, the effect of nonexhaust emissions was more significant than that of exhaust emissions. According to Pant and Harrison (2013) [59], metals can be emitted from various exhaust-related parts, such as fuel/lubricant combustion and engine corrosion, but the level of trace elements emitted in the exhaust is very low and many of these appear most likely to arise from nonexhaust sources. Thus, the load of Zn, Pb, and Cu in the soils due to anthropogenic effects was extracted as the common factor (PC1).

PC2 is responsible for 19.6–22.5% of the total and showed significant positive loadings for CfAs (0.767–0.994) (Figure 2a–c; Table S6). Since As was mostly affected by the lithogenic effect, it was determined that PC2 was the lithogenic factor. PC2 (0.528) and PC1 (0.402) showed a significant positive load for Ni in ARS and GAR. ARS and GAR had a place for vehicle maintenance in common, but the latter was more focused on vehicle storage. Despite Ni concentration in ARS and GAR showing close correlations with Cu or Zn concentrations and its anthropogenic effects being verified, the PCA result led to a different interpretation. In ASY, PC1 and PC2 exhibited a comparable positive load for Ni (0.348 and 0.345, respectively). Since stored or piled vehicles in ASY were expired or malfunctioning, the positive load of PC1 was interpreted as the result of the accumulation of coarse particles, including Ni compounds, from the vehicles on the soil surface. Furthermore, since CfNi for some hotspots found in ASY showed relatively low levels of contamination, PC2 (natural-originated) may have a positive loading for Ni. Nonetheless, PC1 explained the variance of ASY better. Ni in soils for ASY demonstrated that anthropogenic effects were higher than the geological properties.

##### Parking Lot, Driving School, and Roadside

In PAL, DSC, and RDS, the first component (PC1) is responsible for 33.0–43.5% of the total variance and shows significant positive loadings for Zn and Pb (Figure 2d–f; Table S6). These contamination sources are characterized by vehicle stay for a certain period or vehicle movement. The amount of metal produced by tires based on the vehicle running distance data in 2010 in Korea calculated using the German informative inventory report (2012) was 208,739 kg of Zn and 220.94 kg of Pb [78,79], and the amount of harmful components in brake pads in Korea calculated based on the USEPA emission factor was 71.757 kg of Zn and 15,790 kg of Pb [79,80]. Thus, the accumulation of nonexhaust emission of Zn and Pb into the soils may occur due to these contamination sources. In PAL, a vehicle exit and entry cycle is generally shorter compared to that of other sources, while the frequency of using a brake is higher within PAL. Brake wear has been used as the key tracer of nonexhaust particles, including Cu [81,82,83]. The trend of soil deposition of Cu caused by the brake pad friction and tire wear was exhibited in PCA.

The second principal component (PC2) displays 20.3–23.5% of the total variance with high loadings of As (0.509–0.685), Ni (0.726) for PAL, and Cu (0.784, 0.682) for DSC and RDS, respectively (Figure 2d–f; Table S6). According to Gustafsson et al. (2008), the amount of particulate matter generated from vehicles increases with increasing speeds over 30 km h^−1^ [84]. However, high-speed driving rarely occurs in the testing field of DSC; therefore, it was expected that a relatively lower amount of particulates from exhaust gas might be generated from vehicles in DSC than other contamination sources. In RDS, anthropogenic effects caused by Cu were low, because generally, RDS was a place at least 100 m away from the expressway and the frequency of brake use while driving in the highway was relatively lower than that in other roads. The PC2 of the corresponding contamination source explained this trend well.

#### 3.4.2. Cluster Analysis

**Figure 3:**
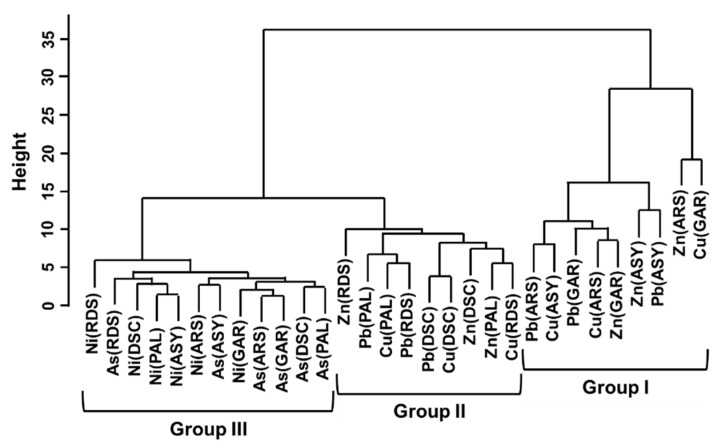
Groups according to the contamination factor (Cf) of metals for each contamination source.

Cfs of metals in all anthropogenic sources were divided into the following three groups: Group I (Zn, Pb, and Cu in ARS, GAR, ASY), Group II (Zn, Pb, and Cu in DSC, RDS, PAL), and Group III (As and Ni in all sources) (Figure 3). For Group I, relatively coarse particles were estimated as the common contaminants during vehicle maintenance or dismantling processes. They accumulated in the soil surface inside or outside the land due to atmospheric events. The contaminants of Group II were the same as those in Group I, but they formed a separate cluster. Group II may contain the impacts of nonexhaust emissions (tire wear) and exhaust emissions while driving, but it formed a cluster closer to Group III. Thus, anthropogenic effects and lithogenic factors were mixed to affect the contamination degrees of Zn, Pb, and Cu in Group II soils. As and Ni (Group III) formed different clusters apart from Group I with anthropogenic properties. In addition, they were grouped with metals of NB for CA in each contamination source (Figure S2a–f). Even though As and Ni were contained in small amounts as parts of automobiles, their lithogenic characteristics were verified again in CA.

## 4. Conclusions

The concentration distribution of Zn, Pb, and Cu near traffic-related facilities was higher than that of NB. The Cf of metals was, in decreasing order, Zn > Pb = Cu > Ni = As. Zn, Pb, and Cu were identified as anthropogenic contaminants and showed significant positive correlations. PCA revealed two contamination causes by metals: coarse particles emitted during the maintenance and dismantling of vehicles in GAR, ARS, and ASY, and nonexhaust/exhaust emissions from driving or parking cars in PAL, DSC, and RDS. Anthropogenic and lithogenic causes were classified as: Group I (Zn, Pb, and Cu in GAR, ARS, and ASY), Group II (Zn, Pb, and Cu in PAL, DSC, RDS), and Group III (As and Ni in all sources). Not only the anthropogenic level of contaminants but also common causes of the soil contamination by metals can be estimated in traffic-related facilities through the combination of the PIs and multivariate analysis. Further study of several potentially contaminated areas will be needed for efficient soil environment management in urban regions affected by anthropogenic activities.

## Figures and Tables

**Table 1:** Distribution of metals in potentially contaminated soils (mg kg^−1^).

Source	*n*	Median	Range	Source	*n*	Median	Range
Zn				Pb			
GAR	283	115.8 ^a^	28.7–1810.9	GAR	283	25.9 ^a^	2.4–642.5
ARS	201	119.2 ^a^	28.4–3372.9	ARS	201	22.7 ^a^	4.5–586.8
ASY	77	112.7 ^ab^	36.5–1255.1	ASY	77	25.1 ^a^	7.9–304.8
PAL	170	94.9 ^b^	23.8–536.7	PAL	170	19.3 ^b^	4.1–231.3
DSC	41	128.1 ^a^	53.2–476.0	DSC	41	31.4 ^a^	8.1–289.0
RDS	29	89.4 ^b^	22.1–246.2	RDS	29	21.0 ^b^	3.5–176.6
NB	88	52	21.3–103.4	NB	88	15.7	4.1–78.3
Cu				As			
GAR	283	23.2 ^ab^	0.5–719.1	GAR	283	4.2 ^a^	0.15–42.57
ARS	201	27.7 ^a^	0.5–489.7	ARS	201	4.0 ^a^	0.75–40.94
ASY	77	29.1 ^a^	0.5–438.3	ASY	77	5.2 ^a^	0.39–45.83
PAL	170	21.3 ^ab^	0.5–165.3	PAL	170	4.0 ^a^	0.35–39.36
DSC	41	28.6 ^ab^	0.5–216.4	DSC	41	2.6 ^a^	0.11–27.79
RDS	29	22.3 ^ab^	0.5–132.5	RDS	29	4.9 ^a^	0.55–22.10
NB	88	13	2.8–50	NB	88	6	0.6–20.6
Ni							
GAR	283	11.0 ^a^	0.2–139				
ARS	201	12.3 ^a^	0.2–90.5				
ASY	77	14.3 ^a^	0.2–67.7				
PAL	170	11.3 ^a^	0.2–63.3				
DSC	41	16.6 ^a^	2.3–40.7				
RDS	29	8.7 ^a^	2.5–37.7				
NB	88	14.4	1.1–114.4				

^a, b,^ and ^ab^ Results of the post-hoc Dunn test (see Table S3). Groups followed by the same character are not significantly different. GAR, garage; ARS, auto repair shop; ASY, auto salvage yard; PAL, parking lot; DSC, driving school; RDS, roadside; NB, natural background.

**Table 2:** Correlation analysis between the contamination factors (Cfs) of metals in contaminated soils by Kendall’s method.

Element	Zn	Pb	Cu	As	Element	Zn	Pb	Cu	As
Garage (GAR)					Auto repair shop (ARS)				
Pb	0.454 ***	-	-	-	Pb	0.481 ***	-	-	-
Cu	0.399 ***	0.445 ***	-	-	Cu	0.441 ***	0.422 ***	-	-
As	0.190 ***	0.210 ***	0.278 ***	-	As	0.130 *	0.222 ***	0.206 ***	-
Ni	0.164 ***	0.169 ***	0.342 ***	0.209 ***	Ni	0.400 **	0.175 ***	0.420 ***	0.237 ***
Auto salvage yard (ASY)					Parking lot (PAL)				
Pb	0.386 ***	-	-	-	Pb	0.349 ***	-	-	-
Cu	0.357 ***	0.435 ***	-	-	Cu	0.368 ***	0.295 ***	-	-
As	−0.013	0.294 ***	0.138	-	As	0.123 *	0.243 ***	0.220 ***	-
Ni	0.168 *	0.328 **	0.389 ***	0.249 **	Ni	0.146 **	0.155 **	0.380 ***	0.159 **
Driving school (DSC)					Roadside (RDS)				
Pb	0.441 ***	-	-	-	Pb	0.123	-	-	-
Cu	0.300 **	0.373 ***	-	-	Cu	0.005	0.379 ***	-	-
As	0.184	0.234 *	0.336 **	-	As	0.135	0.354 **	0.249	-
Ni	0.127	0.236 *	0.337 **	0.248 *	Ni	0.193	0.128	0.069	0.200

*** *p* < 0.005, ** *p* < 0.01, * *p* < 0.05 (significance level).

## Data Availability

Data will be available with permission of the National Institute of Environmental Research (NIER).

## Supplementary Materials

The following are available online at https://www.mdpi.com/article/10.3390/toxics9110278/s1: Figure S1: 801 sampling sites in the study area, including garages (GAR), auto repair shops (ARS), auto salvage yards (ASY), parking lots (PAL), driving schools (DSC), and roadside (RDS); Figure S2: Groups according to metal contamination degree for each contamination source and natural background (NB);Table S1: Summary of the study area; Table S2: Distribution of natural background soil samples in Korea; Table S3: Test of normality involving the concentration of metals in concerned areas (Shapiro–Wilk test); Table S4: Significance of differences in metal concentration for soils in concerned areas; Table S5: Post-hoc Dunn test with characters; Table S6: Kaiser–Meyer–Olkin (KMO) and Fligner–Killeen tests; Table S7: Importance of components for each contamination source; Table S8: Extracted PCs and their rotated loadings.

## Abbreviations

ARS	auto repair shop
ASY	auto salvage yard
CA	cluster analysis
Cf	Contamination factor
DSC	driving school
GAR	garage
NB	natural background (level of soil in Korea)
PAL	parking lot
PCA	principal component analysis
RDS	roadside.
