# Supplementary material for: Identification of Metal Contamination Sources and Evaluation of the Anthropogenic Effects in Soils near Traffic-Related Facilities
Source: Toxics. 2021 Oct 21;9(11):278. doi: 10.3390/toxics9110278 (PMC8618257; doi:10.3390/toxics9110278)
Supplement: Supplementary file 1 [file toxics-09-00278-s001.zip › toxics-1394321-supplementary.pdf]

toxics MDPI

Supplementary Materials:

Identification of Metal Contamination Sources and Evaluation of the Anthropogenic Effects in Soils near Traffic-Related Facilities

Hong-gil Lee, Yoon Joo Byun, Young-Woo Chun, Hoe-Jung Noh, Dong-Jin Kim, Hyun-Koo Kim and Ji-In Kim

Table S1. Test of normality involving the concentration of metals in potentially contaminated areas (Shapiro–Wilk test).

Source	Zn		Pb		Cu		As		Ni	
	w	*p*-value	w	*p*-value	w	*p*-value	w	*p*-value	W	*p*-value
GAR	0.5425	***	0.4457	***	0.4193	***	0.6754	***	0.612	***
ARS	0.3872	***	0.4249	***	0.5603	***	0.6458	***	0.7737	***
PAL	0.7305	***	0.4978	***	0.7848	***	0.7836	***	0.7564	***
ASY	0.5777	***	0.5776	***	0.4908	***	0.6902	***	0.8328	***
DSC	0.8026	***	0.4941	***	0.6372	***	0.7364	***	0.9389	*
RDS	0.8831	**	0.4846	***	0.7131	***	0.8443	***	0.835	***

*** $p < 0.001$, ** $p < 0.01$, * $p < 0.05$ (significance level). GAR, garage; ARS, auto repair shop; ASY, auto salvage yard; PAL, parking lot; DSC, driving school; RDS, roadside; NB, natural background.

Table S2. Significance of differences in metal concentration for soils in potentially contaminated areas.

Element	chi-square	df	p-value
Zn	193.5	5	***
Pb	157.3	5	***
Cu	70.5	5	***
As	50.2	5	***
Ni	80.3	5	***

*** $p < 0.001$ (significance level).

Table S3. Posthoc Dunn test with characters.

Source	Zn	Pb	Cu	As	Ni
GAR	a	a	ab	a	a
ARS	a	a	a	a	a
PAL	b	b	ab	a	a
ASY	ab	a	a	a	a
DSC	a	a	ab	a	a
RDS	b	b	ab	a	a

Groups followed by the same character are not significantly different. GAR, garage; ARS, auto repair shop; ASY, auto salvage yard; PAL, parking lot; DSC, driving school; RDS, roadside; NB, natural background.

Table S4. Kaiser–Meyer–Olkin (KMO) test and Fligner–Killeen test.

Component		GAR	ARS	ASY	PAL	DSC	RDS
KMO value		0.78	0.69	0.60	0.64	0.55	0.54
Fligner–Killeen test	Med chi-square	193.48	124.09	37.335	66.285	22.412	74.983
	df	4	4	4	4	4	4
	p-value	<0.001	<0.001	<0.001	<0.001	<0.001	<0.005

GAR, garage; ARS, auto repair shop; ASY, auto salvage yard; PAL, parking lot; DSC, driving school; RDS, roadside; NB, natural background.

Table S5. Importance of components for each contamination sources.

<GAR>

Component	Standard Deviation	% of Variance	Cumulative %
PC1	1.708	58.3	58.3
PC2	0.990	19.6	77.9
PC3	0.800	12.8	90.7
PC4	0.580	6.7	97.4
PC5	0.361	2.6	100.0

<ARS>

Component	Standard Deviation	% of Variance	Cumulative %
PC1	1.546	47.8	47.8
PC2	1.022	20.9	68.7
PC3	0.944	17.8	86.5
PC4	0.648	8.4	94.9
PC5	0.505	5.1	100.0

<ASY>

Component	Standard Deviation	% of Variance	Cumulative %
PC1	1.597	51.0	51.0
PC2	1.060	22.5	73.5
PC3	0.880	15.5	89.0
PC4	0.628	7.9	96.9
PC5	0.397	3.1	100.0

<PAL>

Component	Standard Deviation	% of Variance	Cumulative %
PC1	1.475	43.5	43.5
PC2	1.084	23.5	67.0
PC3	0.911	16.6	83.6
PC4	0.676	9.1	92.7
PC5	0.603	7.3	100.0

<DSC>

Component	Standard Deviation	% of Variance	Cumulative %
PC1	1.400	39.0	39.0
PC2	1.001	20.3	59.3
PC3	0.983	19.3	78.7
PC4	0.912	16.6	95.3
PC5	0.485	4.7	100.0

<RDS>

Component	Standard Deviation	% of Variance	Cumulative %
PC1	1.284	33.0	33.0
PC2	1.024	21.0	54.0
PC3	0.998	19.9	73.9
PC4	0.911	16.6	90.5
PC5	0.692	9.5	100.0

GAR, garage; ARS, auto repair shop; ASY, auto salvage yard; PAL, parking lot; DSC, driving school; RDS, roadside; NB, natural background.

Table S6. Extracted PCs and their rotated loadings.

<GAR>

Element	Component	
	PC1	PC2
Zn	0.535	−0.055
Pb	0.545	−0.059
Cu	0.495	−0.063
As	0.104	0.994
Ni	0.402	−0.028

<ARS>

Element	Component	
	PC1	PC2
Zn	0.557	−0.186
Pb	0.568	−0.146
Cu	0.540	−0.049
As	0.093	0.815
Ni	0.260	0.528

<ASY>

Element	Component	
	PC1	PC2

<PAL>

Element	Component	
	PC1	PC2

Zn	0.541	−0.376
Pb	0.488	0.141
Cu	0.525	−0.363
As	0.268	0.767
Ni	0.348	0.345

Zn	0.556	−0.299
Pb	0.525	−0.322
Cu	0.555	0.093
As	0.254	0.521
Ni	0.209	0.726

<DSC>

**Element**	**Component**	
	**PC1**	**PC2**
Zn	0.617	−0.301
Pb	0.647	−0.175
Cu	0.204	0.784
As	0.309	0.509
Ni	0.254	−0.073

<RDS>

**Element**	**Component**	
	**PC1**	**PC2**
Zn	0.624	−0.287
Pb	0.633	−0.087
Cu	0.004	0.632
As	0.218	0.685
Ni	0.402	0.205

GAR, garage; ARS, auto repair shop; ASY, auto salvage yard; PAL, parking lot; DSC, driving school; RDS, roadside; NB, natural background.

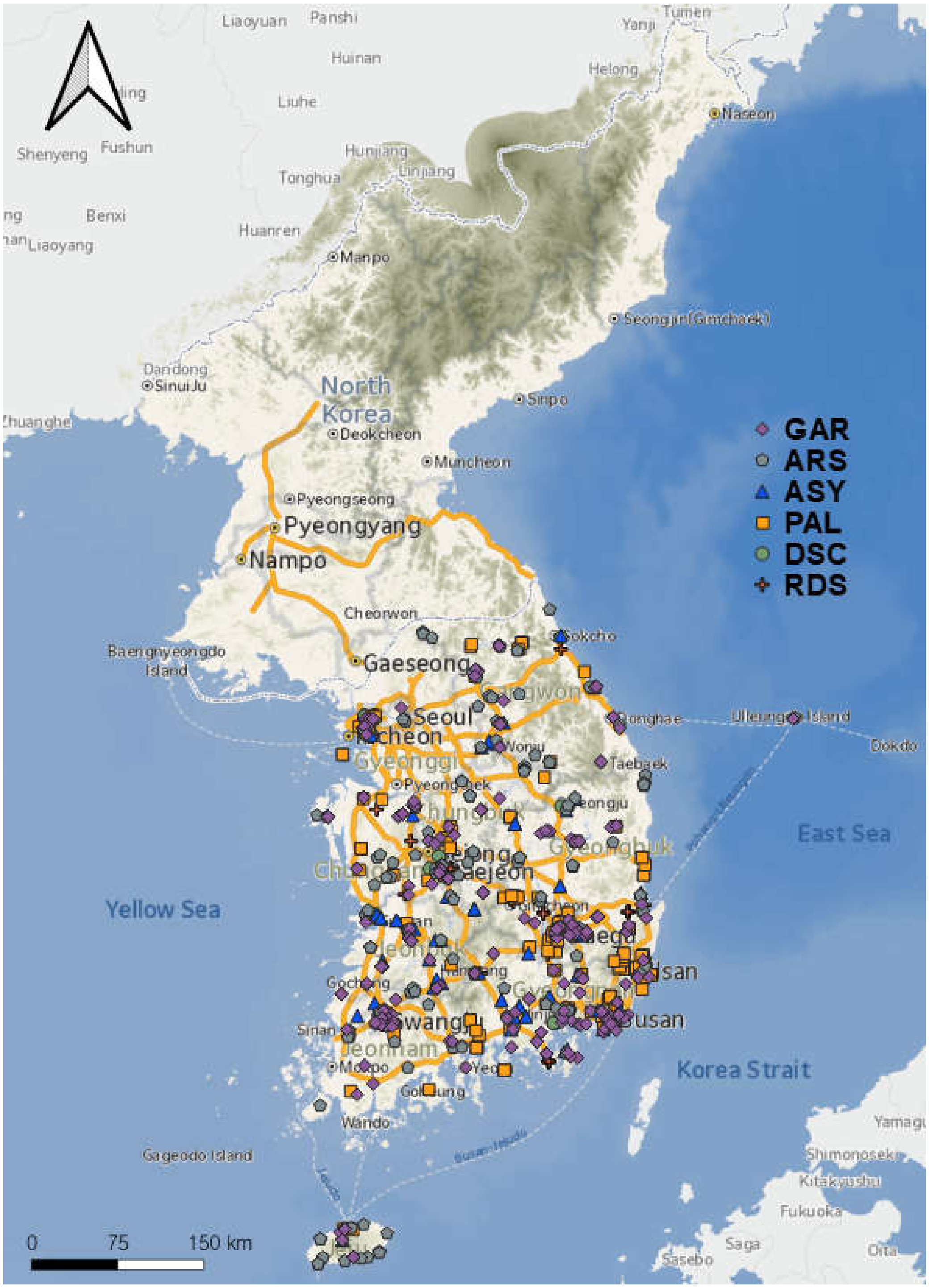

Figure S1. 801 sampling sites in the study area; garage (GAR), auto repair shop (ARS), auto salvage yard (ASY), parking lot (PAL), driving school (DSC), roadside (RDS).

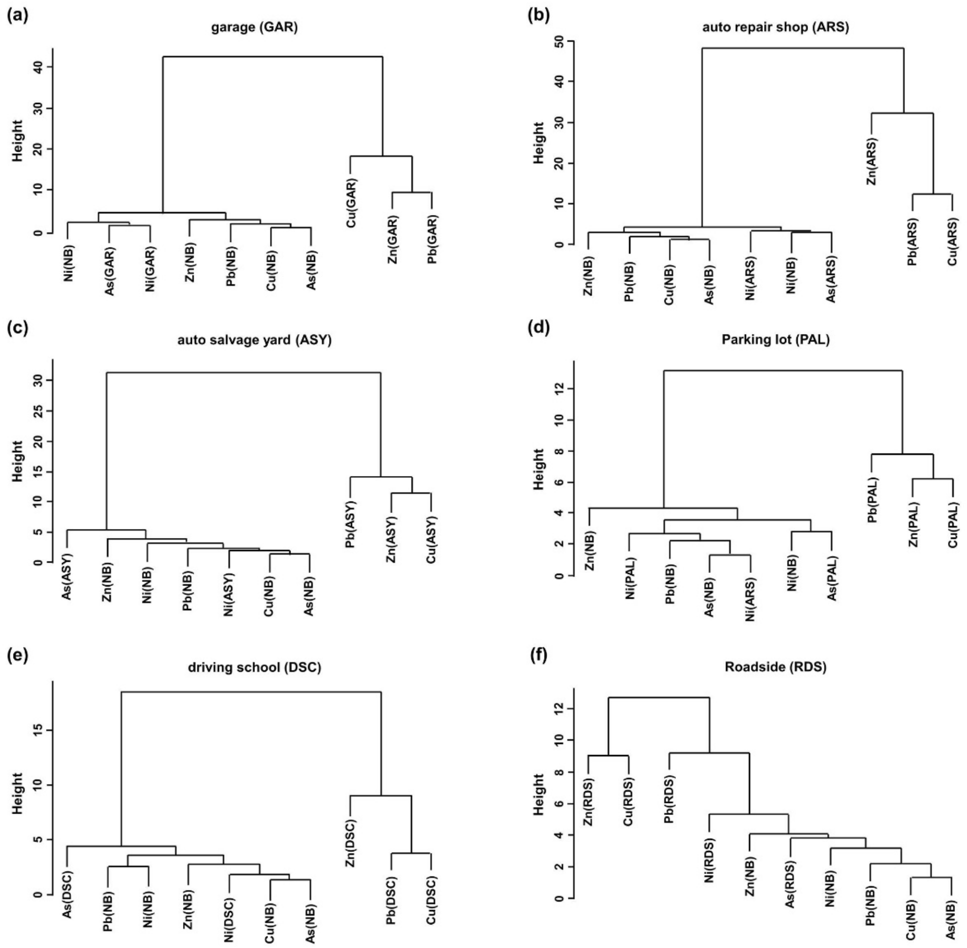

Figure S2. Groups according to metal contamination degree for each contamination source and natural background (NB); (**a**) garage (GAR), (**b**) auto repair shop (ARS), (**c**) auto salvage yard (ASY), (**d**) parking lot (PAL), (**e**) driving school (DSC), and (**f**) roadside (RDS).
